# Targeting drug cocktail hydrogel platform for inhibiting tumor growth and metastasis

**DOI:** 10.1016/j.mtbio.2025.101798

**Published:** 2025-04-23

**Authors:** Liying Xiao, Jianwen Hou, Hongxiang Liu, Qiang Lu

**Affiliations:** a Institutes for Translational Medicine, Soochow University, Suzhou, 215123, People's Republic of China; b National Engineering Laboratory for Modern Silk & Collaborative Innovation Center of Suzhou Nano Science and Technology, Soochow University, Suzhou, 215123, People's Republic of China; c Department of Trauma Orthopedics, The Second People's Hospital of Lianyungang Affiliated to Bengbu Medical College, Lianyungang 222023, People's Republic of China; d Department of Orthopedics, The Second Affiliated Hospital of Soochow University, Soochow University, Suzhou, 215000, People's Republic of China

**Keywords:** Silk nanocarriers, Drug cocktail, Injectable hydrogel, Combination therapy, Tumor recurrence

## Abstract

The combination therapy could overcome the limitation of monotherapy to inhibit tumor recurrence and metastasis, but is usually constrained by complex fabrication processes. Here, a tunable hydrogel platform was developed using different silk nanocarriers, which independently achieve flexible functional optimization of various drugs. Silk nanorods (SNR) were modified with cRGDfK peptides to achieve targeting ability to tumor vessels and then loaded with hydrophobic vascular inhibitor Combretastatin A4 (CA4). The loading of CA4 and the targeted modification could be tuned to enhance the destruction of tumor vessels. Both hydrophilic doxorubicin (DOX) and hydrophobic paclitaxel (PTX) were co-loaded on silk nanofibers (SNF) to form injectable hydrogels with optimized combination chemotherapy. The drug-laden SNR and SNF were blended directly to form injectable hydrogels without the compromise of drug biological activity. Both the targeting modification of SNR and the optimized co-delivery of DOX and PTX improved the therapeutic efficiency *in vitro* and *in vivo*. The long-term inhibition of tumor recurrence and metastasis was achieved through the injectable silk nanocarriers, which are superior to previous combination chemotherapy systems of DOX and PTX. The gradual modular fabrication process and simple physical blending endowed the systems with high flexibility and tunability, suggesting a suitable platform for designing a drug cocktail system.

## Highlights

• Drug cocktail hydrogel platform was developed with different silk nanocarriers for long-term inhibition tumor recurrence and metastasis.
• The platform could load multiple drugs and endow with targeting capacity and responsive ability for synergistic reaction.
• The platform with designable biological functions could meet multiple needs of tumor treatment, bringing optimal therapeutic efficiency.

## Graphical abstract

Drug cocktail hydrogel platform with targeting capacity for long-term inhibition tumor recurrence and metastasis was developed with different silk nanocarriers without the compromise of their respective biological activity.

**Figure:**
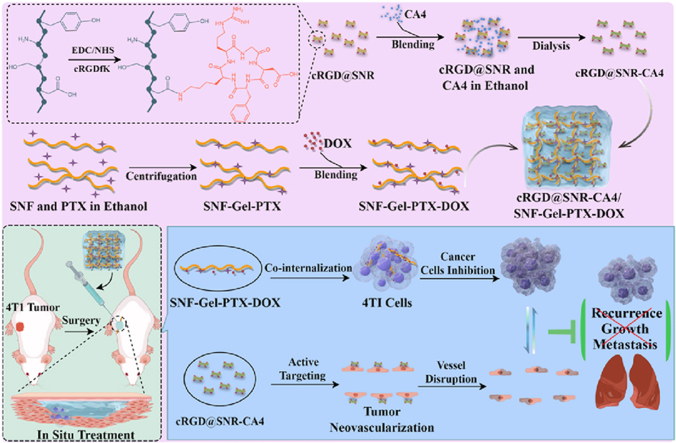

## Teaser

Statement of Significance: Combination therapy is a promising strategy to inhibit tumor recurrence and metastasis, but is limited by complex fabrication processes and inferior optimization of various therapies. Our present work provides a powerful platform for fusing various therapies to optimize the treatment effect. Compared to other reported combination therapy strategies, the present hydrogel platform showed better controllability and designability. Besides the co-loading of multiple drugs, more functions, such as targeting capacity and responsive ability, could be introduced easily to improve the synergistic reaction of various cargos. The excellent inhibition of both tumor growth and metastasis confirmed the advantages of the hydrogel system. Our present study makes it possible to design specific biological functions based on multiple needs of tumor treatment, which has a promising future in combination therapy with optimal therapeutic efficiency.

## 1 Introduction

Due to the complexity, diversity, and heterogeneity of tumors, the patients treated with monotherapy usually suffer from recurrences and metastasis [1]. To overcome the impediments to monotherapy, increasing studies are focusing on combination therapy to optimize the synergistic action of multiple therapeutics and therapies, improving therapeutic efficacy [[2], [3], [4], [5], [6], [7], [8], [9]]. Several challenges remain for the combination therapy, such as the complexity of combining different therapies and the lack of programmable control for multiple drugs and therapies. For example, to improve synergistic effects, some anti-tumor drugs with distinctly different hydrophilic and hydrophobic properties tend to deliver into cancer cells simultaneously, which demands co-loading and immobilization of multiple drugs on the same carriers [10,11]. At the same time, other agents with various biological functions prefer to act on different cells and avoid phagocytosis by tumor cells [8,[12], [13], [14]]. Infusing different carriers into the same delivery platform is a promising option to develop a smart delivery system used in combination therapy, but unfortunately seems unattainable for most delivery systems.

Injectable hydrogels are reasonable platforms in tumor combination therapy due to their marked properties, such as efficient encapsulation of therapeutic cargos, ease of fabrication, and good shape adaptability [15]. Multiple hydrophilic and hydrophobic drugs with distinct biological functions have been loaded inside injectable hydrogels and achieved sustained co-delivery to inhibit tumor growth and metastasis [13,16]. Different therapy options, such as chemotherapy, radiotherapy, immunotherapy, and gene therapy, were also introduced to a single injectable hydrogel to improve therapeutic efficacy against tumors [15,[17], [18], [19]]. The hydrogels could be modified to achieve stimuli-responsive capacity or targeted ability, which brought smart delivery of therapeutic agents and better tumor suppression [20,21]. However, the major strategy in these injectable hydrogel systems focused on the delivery control of bioactive cargos rather than the internalization behavior of different cells. More active delivery tactics that could regulate the ingress of various drugs into specific cells would be beneficial to better combination therapy. Unlike hydrogels, nanocarriers with a suitable size and morphology could be swallowed directly by cells [22]. Several therapeutic molecules with distinct physical and chemical properties have been immobilized in a single nanocarrier and enter tumor cells simultaneously, exhibiting a better synergistic effect than when they were co-delivered from different nanocarriers [10,11,23]. The drug-laden nanocarriers could be decorated to obtain targeting characteristics to specific cells, which further enhanced therapeutic efficiency against cancers [5,14,24,25]. Thus, injectable hydrogels composed of nanocarriers are a preferable option to fuse both advantages of injectable hydrogels and nanocarriers. However, few nanocarriers could form injectable hydrogels with the ability to integrate multiple drugs with optimal therapeutic effect.

**Scheme 1:**
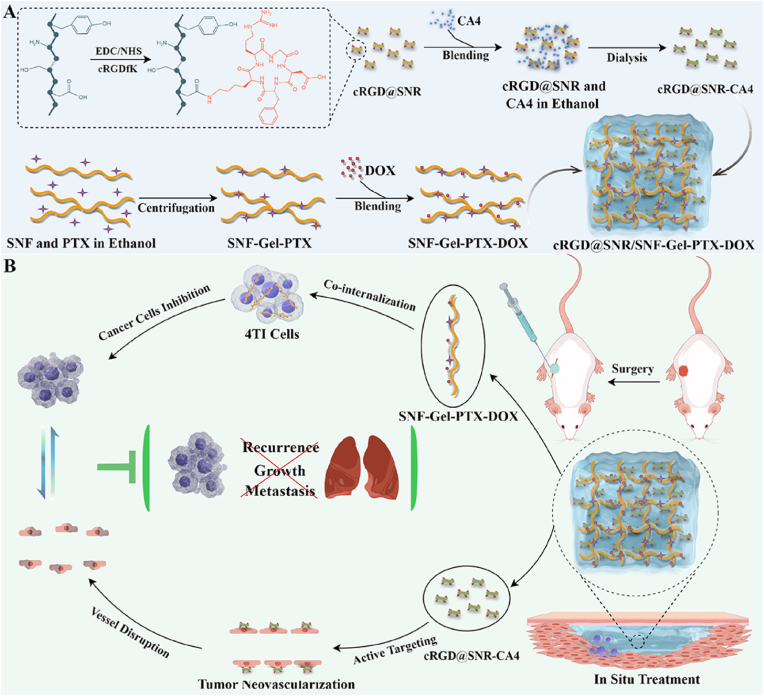
Schematic illustration of the targeting injectable drug cocktail hydrogel platform for inhibiting tumor growth and metastasis. (By Figdraw).

Recently, shear-thinning silk hydrogels as injectable carriers were used to treat cancers through combination therapy, including chemo/photothermal/photodynamic therapies [13,16,[26], [27], [28], [29], [30]]. Beta-sheet-rich silk nanofibers (SNF) were assembled in an aqueous solution and exhibited enhanced loading and release performances for both hydrophobic and hydrophilic drugs [31,32]. Similar to other silk hydrogels [13,16,26,27], drug-laden SNF assembled into injectable hydrogels [23,32], facilitating its applications as nanocarriers. The size and morphology of the SNF can be tuned through simple physical processes to enhance cell internalization without sacrificing loading and release performances [22]. Chemical modification was also feasible based on phenol, carboxylic, and hydroxyl groups [33,34], making silk nanocarriers promising candidates to achieve combination therapy with smart delivery capacity. Doxorubicin (DOX) and paclitaxel (PTX) co-load SNF form injectable hydrogels, enhancing local combination chemotherapy [23]. The synergistic antitumor effect of angiogenesis inhibitor Combretastatin A4 (CA4) and chemotherapeutic cytotoxicity (DOX/PTX) was anticipated to further inhibit tumor recurrence and metastasis [[35], [36], [37]]. Here, as a proof of concept, beta-sheet rich SNF (1–2 μm) were cut to silk nanorods (SNR) with a length of below 100 nm, facilitating cell internalization. The SNRs were modified with peptides to bring targeting capacity to tumor neovascularization endothelial cells and then loaded with hydrophobic Combretastatin A4 (CA4) through the co-solvent method (Scheme 1). The targeted drug-laden SNR were then blended with SNF co-loaded with DOX and PTX, forming injectable drug cocktail hydrogels. In this hydrogel system, the SNF could deliver both DOX and PTX to the same cancer cells simultaneously to improve combination chemotherapy efficiency, while the SNR transferred the drugs to tumor neovascularization specifically to inhibit the metastasis. Both *in vitro* and *in vivo* results revealed that the drug-laden SNF and SNR exhibited their anticipated biological function, achieving enhanced combination therapy. The long-term inhibition of tumors, as well as minimal metastasis, was achieved for the drug cocktail hydrogel, which implied its promising future in cancer treatment.

## 2 Materials and methods

### 2.1 Fabrication of SNF and SNR

The reported concentration-dilution-incubation processes were used to fabricate SNF [38]. Simply, the regenerated silk solutions prepared via traditional lithium bromide procedures were concentrated to over 20 wt%, diluted with deionized water to 20 mg/mL and 5 mg/mL, and then incubated at 60 °C until hydrogelation, following SNF formation. The SNF hydrogels were termed SNF-Gel. SNF (5 mg/mL) was treated by an ultrasonic homogenizer (JY92-IIN, Scientz Biotechnology Co., Ningbo, China) to obtain SNR with a length of about 80 nm [22]. The treatment conditions were as follows: ultrasonic intensity 650 W, sonication period 3.5 s, pause period 1.5 s, treatment time 30 min.

### 2.2 Synthesis of cRGD@SNR

1-ethyl-(3-dimethylaminopropyl) carbodiimide hydrochloride (EDC) and N-hydroxysuccinimide (NHS) were used to crosslink cRGDfK peptide on the surface of SNR to induce targeting ability [24]. The SNR (20 mL, 2.5 mg/mL) was activated by adding 30 mg NHS and 40 mg EDC, followed by stirring at room temperature for 30 min. Subsequently, cRGDfK (0.1 mL, 5 mg/mL) was added to the above solution and stirred for 24 h at room temperature. The conjugated cRGD@SNR was dialyzed (MWCO, 3500 Da) against deionized water for 3 days to remove excess coupling reagents and free cRGDfK. The obtained samples were termed as cRGD@SNR.

### 2.3 Preparation of SNR-CA4 and cRGD@SNR-CA4

CA4 (Yuanye, Shanghai, China) was loaded on SNR and cRGD@SNR through a simple blending-dialyzing process [22]. CA4 was dissolved in ethanol and blended with SNR and cRGD@SNR at a volume ratio of 1:1 at room temperature for 20 h, followed by dialysis (MWCO, 3500 Da) to remove the unloaded CA4. The concentration of CA4 outside the dialysis tube was measured via ultraviolet–visible (UV–vis) spectrometer (Cary5000, Agilent, Santa Clara, CA, U.S.A.) at 305 nm. The loading capacity (LC%) and loading efficiency (LE%) were calculated using the traditional formulas [22]. The CA4-laden SNR and cRGD@SNR were labeled as SNR-CA4 and cRGD@SNR-CA4, respectively.

### 2.4 Characterization of the SNRs

The morphology of SNR, cRGD@SNR, SNR-CA4, and cRGD@SNR-CA4 was observed using an atomic force microscope (AFM, Nanoscope V, Veeco, NY, U.S.A.) in air. The height of SNRs was analyzed using NanoScope Analysis software, while the width distribution of the samples based on AFM images was analyzed using ImageJ software. FTIR spectra were determined in a Nicolet FTIR 5700 spectrometer (Thermo Scientific, FL, U.S.A.). Freeze-dried SNR and cRGD@SNR were dissolved in deuterium oxide (D_2_O) containing 9.3 M LiBr, and ^1^H NMR spectra were determined in an AVANCE NEO NMR system at 400 MHz (Bruker, Karlsruhe, Germany). The zeta potential was measured by a Zetasizer (Nano ZS, Malvern, Worcestershire, UK) at 25 °C.

### 2.5 Preparation of SNF-Gel-PTX-DOX and injectable drug cocktail hydrogel platform

The PTX-DOX-co-laden SNF was prepared through our previous loading processes [23]. Simply, hydrophobic PTX (Yuanye, Shanghai, China) was loaded on SNF and dispersed in water using an organic solvent-water system. Then, DOX (Yuanye, Shanghai, China) was added to the PTX-laden SNF hydrogel directly, and laden on the SNFs directly. The PTX-DOX-co-laden SNF hydrogel was termed SNF-Gel-PTX-DOX. SNR-CA4 and cRGD@SNR-CA4 were mixed with SNF-Gel-PTX-DOX (1:1 v/v) directly to obtain an injectable drug cocktail hydrogel platform, and termed SNR-CA4/SNF-Gel-PTX-DOX and cRGD@SNR-CA4/SNF-Gel-PTX-DOX, respectively. The rheological property of the injectable drug cocktail hydrogel was determined by rheometry (DHR-2, TA Instruments, New Castle, U.S.A) at 37 ± 1 °C. The hydrogels were subjected to a sustained strain of 1 % for 120 s, alternating with a sustained strain of 100 % for 30 s for at least three cycles with a frequency of 10 rad/s.

### 2.6 Cell culture

The mouse breast cancer cells (4T1) and human umbilical vein vascular endothelial cells (HUVEC) were purchased from Shanghai Cell Bank of the Chinese Academy of Sciences. The cells were cultured with Dulbecco's modified Eagle medium (DMEM, Gibco, BRL, MD, U.S.A) containing 10 % fetal bovine serum (FBS, Gibco, BRL, MD, U.S.A) and 1 % penicillin-streptomycin (Invitrogen, Grand Island, NY, U.S.A) in a 5 % CO_2_ cell incubator at 37 °C.

### 2.7 Cytotoxicity test

4T1 and HUVEC cells (3 × 10^3^ cells/well) were inoculated into 96-well plates and cultured overnight for cell recovery. The cells were cultured in media containing cRGD@SNR, CA4, SNR-CA4, and cRGD@SNR-CA4 for 48 h, respectively. The concentration of PTX, DOX, and CA4 was 50 nM, 100 nM, and 1000 nM, respectively. The cell morphology was observed by an inverted fluorescence microscope (Axio Vert A1, Carl Zeiss, Germany). The cell viability was measured using Cell Counting Kit 8 (10 %, CCK-8, Beyotime, Shanghai, China). Similarly, the cell cytotoxicity of SNF-Gel-PTX-DOX, CA4/PTX/DOX, CA4/SNF-Gel-PTX-DOX, and cRGD@SNR-CA4/SNF-Gel-PTX-DOX to 4T1 cells was evaluated according to the above methods. To reveal the synergistic interactions of PTX and DOX, the viability of 4T1 cells was measured when incubated with free PTX/DOX and SNF-Gel-PTX-DOX with different combinations of PTX and DOX (4:1, 2:1, 1:1, 1:2, and 1:4) for 48 h. The total drug concentration ranged from 1 to 10000 nM. The cell viability was measured using CCK-8.

### 2.8 In vitro β-tubulin polymerization restraint

HUVECs were inoculated into the 14 mm cell slivers in a 24-well plate at a density of 2 × 10^4^ cells/well. The cells were cultured in media containing cRGD@SNR, CA4, SNR-CA4 and cRGD@SNR-CA4 as well as SNF-Gel-PTX-DOX, CA4/PTX/DOX, CA4/SNF-Gel-PTX-DOX and cRGD@SNR-CA4/SNF-Gel-PTX-DOX for 48 h. The concentration of PTX, DOX, and CA4 was 50 nM, 100 nM, and 1000 nM, respectively. After fixed with 4 % paraformaldehyde, blocked with 3 % Bull Serum Albumin (BSA), and permeabilized with 0.2 % Triton X-100 (in 1 % BSA solution), the cells were incubated with mouse monoclonal antibodies against β-tubulin (1:200 dilution, 3 % BSA solution) overnight. Then, the cells were incubated with FITC-labeled secondary antibody (anti-mouse antibody, 1:1000, in PBS) for 2 h at 37 °C, stained with Diamidinyl Phenyl Indole (DAPI) for 5 min. Finally, the cells were imaged with confocal laser scanning microscopy (CLSM, TCS SP8, Leica, Germany).

### 2.9 In vivo tumor neovascularization regression

Female BALB/c nude mice (3–5 weeks) were provided by the Experimental Animal Research Center of Soochow University, and procedures strictly complied with the ethical guidelines for experimental animals in Soochow University (202101A252). 4T1 cells (1 × 10^6^ cells/mL) were resuspended in PBS and high protein membrane (1:1 v/v). 50 μL of cell suspension was injected into the groin of mice with an insulin syringe. When the tumor size reached 100 mm^3^, 100 μL of normal saline (Control group), cRGD@SNR, CA4, SNR-CA4, and cRGD@SNR-CA4 were injected. The dosage of CA4 was 2.5 mg/kg for the drug administration groups. After 2 days, the mice were euthanized, and the hemorrhage case of the tumor was observed. The tumors were fixed in 10 % formalin buffer, and then dehydrated, paraffin-embedded, and sectioned to obtain the section samples. The sections were stained with hematoxylin-eosin (H&E) and platelet endothelial cell adhesion molecule-1 (CD31). The stained samples were imaged using an inverted fluorescence microscope (Axio Vert A1, Carl Zeiss, Germany).

### 2.10 In vivo tumor recurrence and metastasis

Female BALB/c mice (3–5 weeks) were provided by the Experimental Animal Research Center of Soochow University, and procedures strictly complied with the ethical guidelines for experimental animals in Soochow University (202110A0164). 4T1 cells (1 × 10^6^ cells/mL) were resuspended in PBS and high protein membrane (1:1 v/v). 100 μL of cell suspension was injected into the groin of mice with an insulin syringe. Tumor resection was performed when the tumors reached 200 mm^3^. 3 days after surgery, the mice were randomly divided into five groups (n = 5): Control, cRGD@SNR/SNF-Gel, SNF-Gel-PTX-DOX, SNR-CA4/SNF-Gel-PTX-DOX, and cRGD@SNR-CA4/SNF-Gel-PTX-DOX, and the time point was set as day 0. 200 μL of samples were injected into the postoperative cavity, while 200 μL of normal saline was injected into the control group. The dosages of PTX, DOX, and CA4 were 2.5 mg/kg, 5 mg/kg, and 2.5 mg/kg, respectively. The body weight of mice and the volume of the tumors were monitored every 4 days.

The volume (V) of the tumor was calculated according to the formula:

$$V=\left({\text{ab}}^{2}\right)\;/2$$

Where a and b were the maximum and minimum diameters of the tumors. On day 20, the mice were euthanized.

The tumors, hearts, and lungs were fixed, dehydrated, paraffin-embedded, and sectioned. The sections were stained with H&E and Terminal Deoxynucleotidyl Transferase Nick and Labeling (TUNEL). The samples were also immunostained against Ki-67 and CD31. The samples were then imaged using an inverted fluorescence microscope (Axio Vert A1, Carl Zeiss, Germany).

### 2.11 Statistical analysis

Data were presented as mean ± standard deviation (SD). Student's t-test with two-sided method was used to evaluate p-values. One-way ANOVA combined with Bonferroni's multiple comparison Post Hoc test was used to evaluate statistical analysis among multiple groups. ns > 0.05 indicated that the results were not statistically significant. ∗P ≤ 0.05, ∗∗P ≤ 0.01, ∗∗∗P ≤ 0.001 indicated that the results were statistically significant.

## 3 Results and discussion

### 3.1 Targeting modification of SNRs

**Fig. 1:**
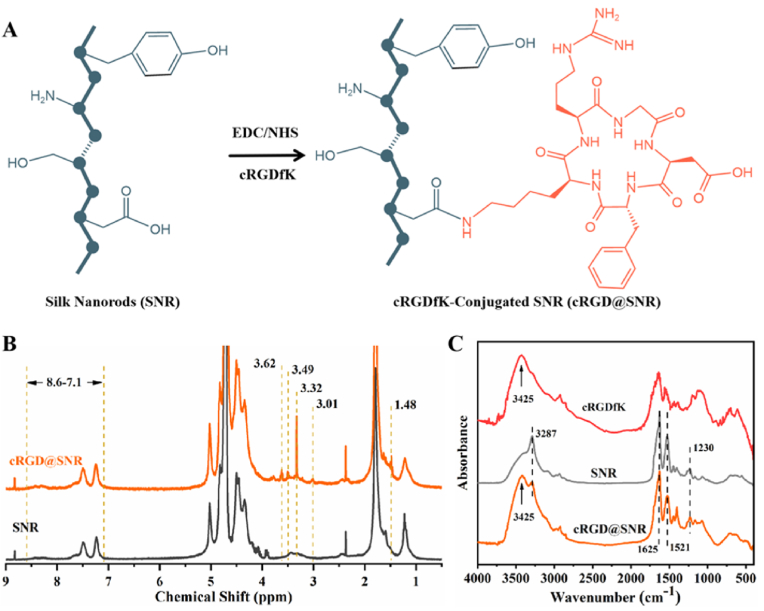
(A) Synthetic route of the cRGDfK conjugated SNR (cRGD@SNR). (B) ^1^H NMR spectra of SNR and cRGD@SNR. The peak at 4.72 ppm was attributed to the solvent (D_2_O). The benzene ring group attributed the peaks between 7.1 and 8.6 ppm. (C) FTIR spectra of cRGDfK, SNR and cRGD@SNR.

Our recent study revealed that SNF (length 1–2 μm) could transform into SNR with a length of about 80 nm after ultrasonic treatment without compromise of loading capacity for both hydrophilic and hydrophobic drugs [22]. The drug-laden SNR exhibited better cell internalization than SNF, resulting in more effective tumor inhibition [22]. Considering the high density of carboxylic groups on SNR, EDC and NHS were used to cross-link cRGDfK peptide on SNR to induce targeting ability on tumor neovascularization endothelial cells (Fig. 1A). After chemical modification, the zeta potential of SNR decreased from −48 mV to −36 mV, an indicator of carboxylic loss. Compared to that of unmodified SNR, new peaks corresponding to the typical peaks of cRGDfK [25], appeared in the ^1^H NMR spectra of the modified SNR (cRGD@SNR) (Fig. 1B and Fig. S1-S2). The results suggested the cRGDfK molecules have been successfully conjugated with SNR through the carboxylic group, implying their targeting capacity. In the FTIR spectra, the characteristic peak at 3425 cm^−1^ in the curve of cRGD@SNR corresponded to the aldimines group in the cyclic structure of cRGDfK (Fig. 1C). FTIR spectra revealed that SNR maintained typical peaks of beta-sheet structures (3287 cm^−1^, 1625 cm^−1^, 1521 cm^−1^, and 1230 cm^−1^) after chemical modification. Thus, SNR with targeting ability on endothelial cells was developed without the changes in secondary structures.

**Fig. 2:**
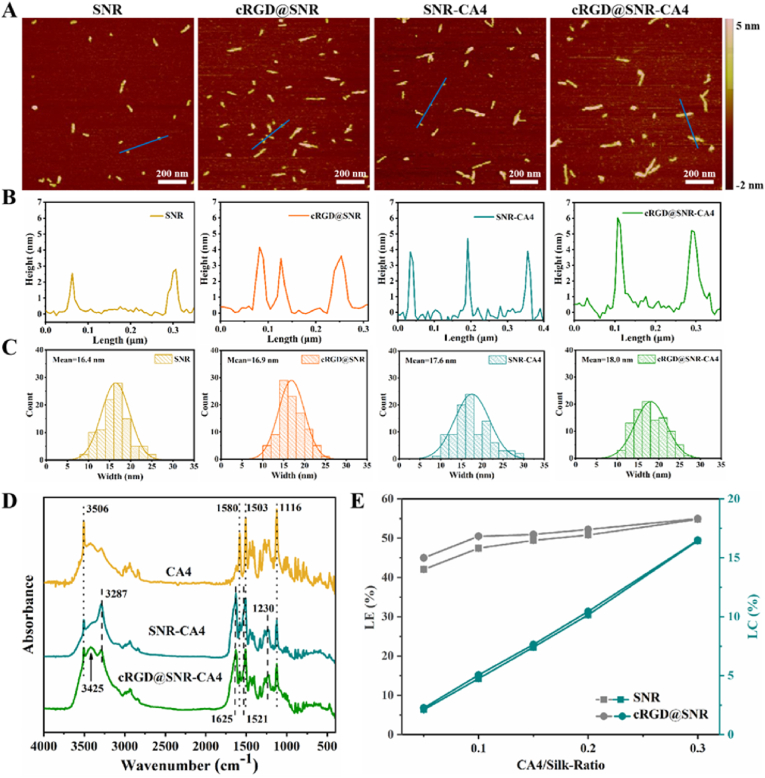
(A) AFM images, (B) the height profile of the AFM images, (C) the width distribution of the nanocarriers based on AFM images analyzed using ImageJ software (n = 100). (D) FTIR spectra of CA4, SNR-CAE, and cRGD@SNR-CA4. (E) The loading efficiency (LE%) and loading capacity (LC%) of CA4 for SNR and cRGD@SNR at different CA4/silk ratios. Data were presented as mean ± SD, n = 3, error bars indicate the SD, One-way ANOVA combined with Bonferroni's multiple comparison Post Hoc test was used to evaluate statistical analysis. There was no statistically significant difference between the SNR and cRGD@SNR group. The samples were as follows: SNR are silk nanorods, cRGD@SNR are cRGDfK-conjugated SNR, SNR-CA4 are CA4-laden SNR, and cRGD@SNR-CA4 are CA4-laden cRGD@SNR.

The beta-sheet-rich structure of SNR favored the loading of hydrophobic drugs [31]. CA4 is an angiogenesis inhibitor and has been used as an anti-cancer drug [12]. To improve its bioavailability, multiple nanocarriers have been designed to immobilize and disperse CA4 in aqueous environments, where complex fabrication processes were required [12,14,[39], [40], [41]]. SNR could load and disperse CA4 in aqueous solutions through a simple blending-dialyzing process. The morphology of SNRs was observed using AFM (Fig. 2A). Both the width and thickness of SNRs increased after 4 % of CA4 was loaded, while all SNRs were well dispersed and rod-shaped (Fig. 2B and C). In the FTIR spectra, the typical peaks of CA4 (3506 cm^−1^, 1580 cm^−1^, 1503 cm^−1^, and 1116 cm^−1^) appeared following the introduction of CA4 to the SNR and cRGD@SNR, revealing the successful loading of CA4 (Fig. 2D). After the introduction of CA4, both SNR and cRGD@SNR maintained beta-sheet rich structures, suggesting the stability of the nanocarriers. Similar to SNR, the loading efficiency (LE%) and loading capacity (LC%) were 55.00 % ± 0.05 % and 16.50 % ± 0.02 % for cRGD@SNR, respectively (Fig. 2E). The results revealed that the chemical modification of cRGDfK had no negative influence on the loading capacity of SNRs. Sufficient CA4 was loaded on the SNR with targeting ability, which would favor the targeted vascular inhibition in tumor tissues.

**Fig. 3:**
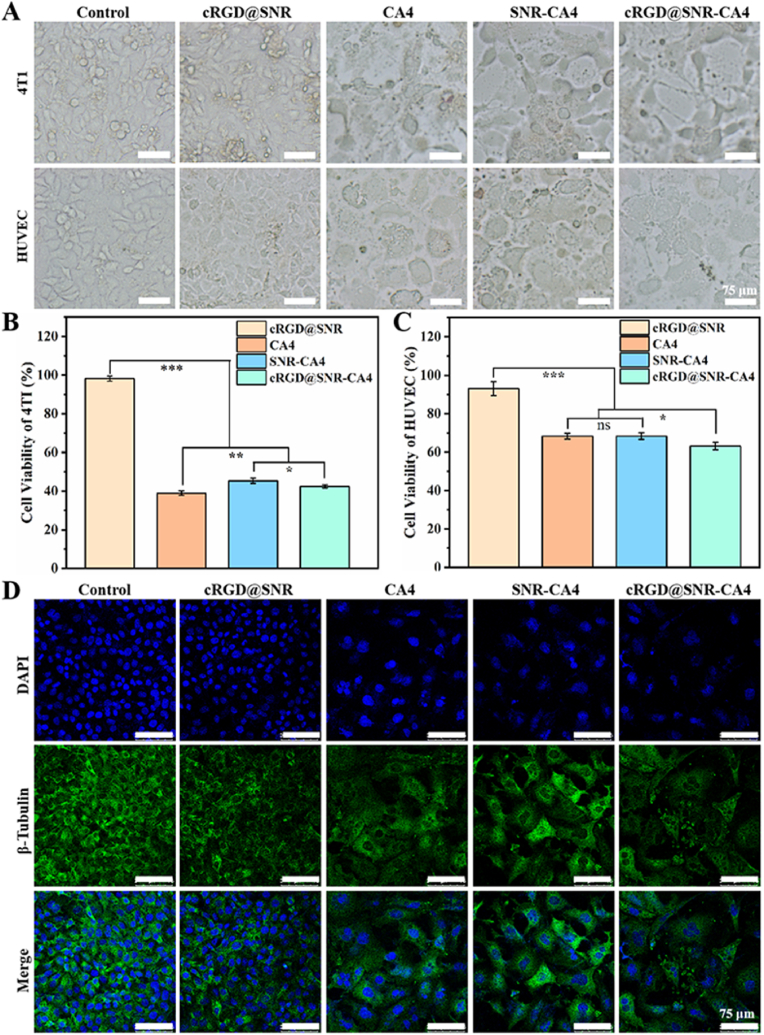
Cell morphology (A) and cell viability of 4T1 cells (B) and HUVEC cells (C) incubated with samples for 48 h. (D) Immunofluorescence staining of β-Tubulin in HUVECs after incubation with samples for 48 h. Untreated cells were considered as control. DNA stained with DAPI (blue) and β-Tubulin marked in green. The samples were cRGD@SNR, CA4, SNR-CA4 and cRGD@SNR-CA4. Data presented as mean ± SD, n = 5, error bars indicate the SD, p-values were calculated using Student's *t*-test with two-sided method, ns > 0.05, ∗P ≤ 0.05, ∗∗P ≤ 0.01, ∗∗∗P ≤ 0.001. (For interpretation of the references to colour in this figure legend, the reader is referred to the Web version of this article.)

To evaluate the influence of targeting modification of SNR on the therapeutic efficiency of CA4, free CA4, CA4-laden SNR, and cRGD@SNR-CA4 were cultured with 4T1 cells and endothelial cells (Fig. 3). The cells were also cultured in the medium containing CA4-free cRGD@SNR to reveal the influence of the immobilized cRGDfK. Compared to the control group (plate group), similar viability of both 4T1 cells and endothelial cells was achieved for the control and cRGD@SNR group, suggesting that the immobilized cRGDfK never declined the cytocompatibility of SNR (Fig. 3A–C). CA4 has been used to treat cancers due to its toxicity to both tumor cells and endothelial cells [42]. Due to higher phagocytosis of cancer cells [22], both free and loaded CA4 exhibited higher cytotoxicity to 4T1 cells when cultured for 48 h (Fig. 3B and C). Free CA4 was more cytotoxic than that loaded on SNR and cRGD@SNR, which was consistent with previously reported studies [23,31]. For endothelial cells, significantly higher cytotoxicity was observed in the cRGD@SNR-CA4 group, indicating the targeting ability of the immobilized peptides (Fig. 3A–C). CA4 restrains the β-tubulin polymerization to inhibit tumor angiogenesis [43]. To further evaluate the targeting function of the cRGD@SNR, the β-tubulin in HUVECs was stained with green and measured with a confocal microscope (Fig. 3D). When the HUVECs were cultured for 48 h in different groups, strong green fluorescence appeared in the control and CA4-free cRGD@SNR group, indicating normal β-tubulin polymerization. All the free CA4 and CA4-laden SNR groups showed significantly lower fluorescence intensity, which confirmed the bioactivity of CA4. When the same amount of CA4 was loaded on SNR and cRGD@SNR, the secretion of β-tubulin was effectively restrained in the cRGD@SNR group due to the targeting effect of cRGDfK, suggesting the good targeting ability.

**Fig. 4:**
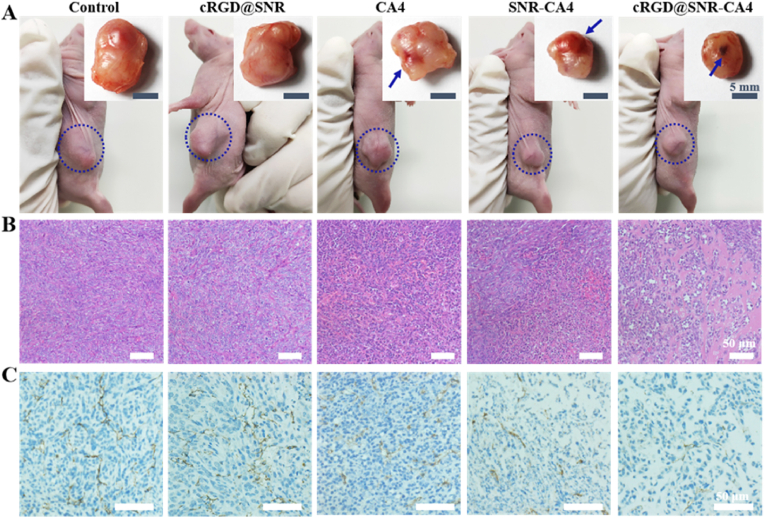
The photographs (A), H&E staining (B), and CD31 immunohistochemical staining (C) of harvested tumors after cRGD@SNR, CA4, SNR-CA4, and cRGD@SNR-CA4 were injected into the tumor in 4T1 tumor-bearing mice for 48 h. In Figure A, the blue arrows indicate the hemorrhage sites. In Figure C, brown indicates endothelial cells of tumor vessels, and blue indicates the cell nucleus stained with hematoxylin. (For interpretation of the references to colour in this figure legend, the reader is referred to the Web version of this article.)

Our recent study revealed that SNR had no toxicity to tumor tissues [22]. Considering that SNR and cRGD@SNR showed similar cytocompatibility *in vitro*, only cRGD@SNR was injected into the tumor in rats and compared with CA4-laden SNR. Similar to the PBS group, the tumor with rich angiogenic networks was maintained in the cRGD@SNR group, indicating that CA4-free SNR never restrained tumor growth (Fig. 4). CA4 induces tumor necrosis through inhibiting tumor neovascularization and causing hemorrhage [42]. Several local hemorrhages appeared at the tumor site when free CA4 solution was injected into tumor tissue (Fig. 4A). Although slight higher cytotoxicity was observed in free CA4 group than in SNR-CA4 group *in vitro*, more hemorrhage area and smaller tumor size were found in the SNR-CA4 group, indicating better biological function of CA4 loaded on the SNR (Fig. 4A). Drug-laden SNR could stay in tumor tissue to provide long-term effect [22]. Similar to previous studies [22], CA4 was also retained in the tumor through SNR, avoiding the quick loss of free CA4. The targeting modification of SNR further enhanced the action of CA4. The CA4 loaded on the cRGD@SNR preferred to act on the tumor vessels, resulting in more serious regression of neovascularization and hemorrhage (Fig. 4A–C). Significant lower density of tumor cells was found in the tumor treated with cRGD@SNR-CA4, an indicator of effective tumor necrosis (Fig. 4B). Both *in vitro* and *in vivo* results clarified that the cRGDfK-modified SNR could load and disperse hydrophobic CA4 in aqueous solution and target the tumor vessels to induce hemorrhage and restrain tumor growth. However, the targeted monotherapy based on CA4-laden SNR cannot long-term inhibit tumor growth effectively. More chemotherapy is required to fuse with CA4-laden SNR to achieve the combination therapy.

### 3.2 Combination chemotherapy optimization based on SNF hydrogel

**Fig. 5:**
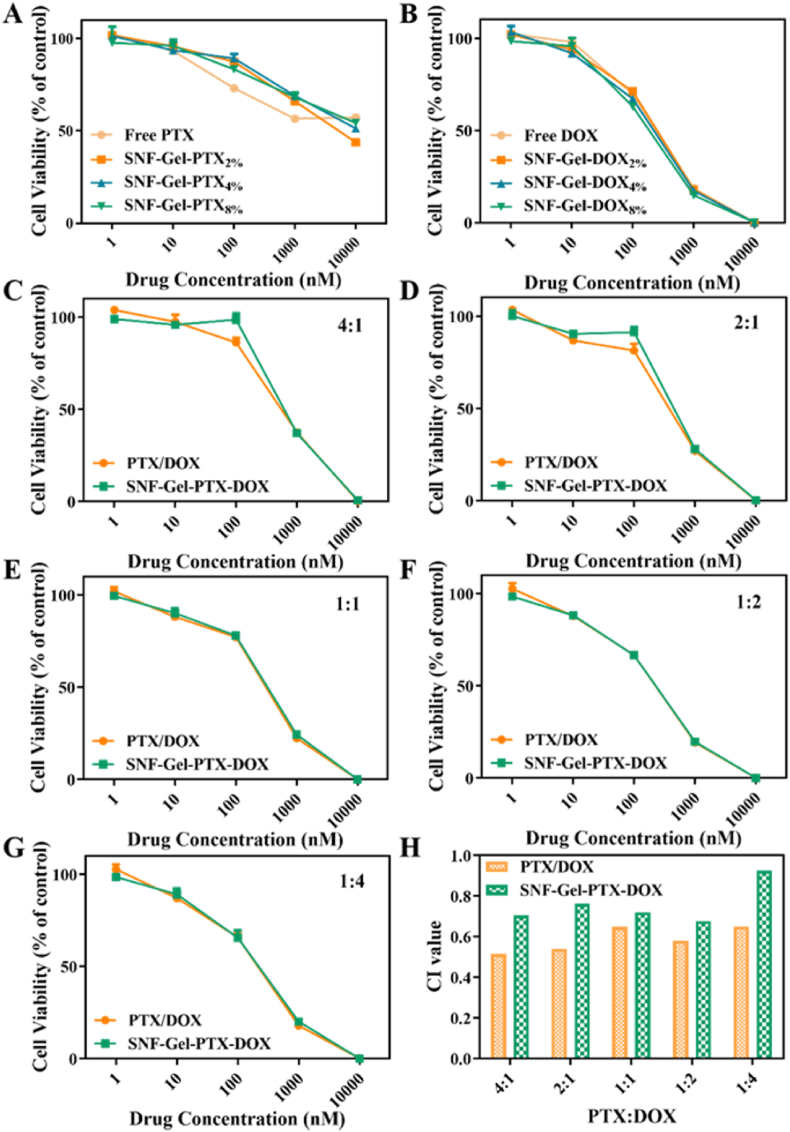
Cell viability of 4T1 cells against the PTX-laden SNF (A) and DOX-laden SNF (B) with different loading concentrations for 48 h. (C–G) Cell viability of 4T1 cells against Free PTX/DOX and SNF-Gel-PTX-DOX with treatment combinations of PTX and DOX at 4:1 (C), 2:1 (D), 1:1 (E), 1:2 (F), and 1:4 (G) for 48 h. (H) CI values (50 % cell viability inhibition) of Free PTX/DOX and SNF-Gel-PTX-DOX with different treatment combinations of PTX and DOX (4:1,2:1,1:1,1:2, and 1:4) against 4T1 cells for 48 h. Data presented as mean ± SD, n = 5, error bars indicate the SD.

SNF was used to co-load DOX and PTX simultaneously and form injectable hydrogels to enhance combination chemotherapy [23]. Some DOX was released slowly from the hydrogels and then engulfed by cancer cells, while the other DOX and all PTX were immobilized on the same silk nanofibers and co-internalized by cancer cells. Since both hydrophilic and hydrophobic drugs (DOX, PTX) loaded with silk nanocarriers played its roles through cellular internalization rather than the release, the combination chemotherapy was directly evaluated based on cellular behaviors. The ratio and concentration of DOX and PTX were easily tuned in the delivery system to optimize the combination chemotherapy, which strengthened its applications in various tumors. To reveal the optimal ratio and concentration of DOX and PTX co-loaded on SNF for 4T1 cells, the PTX-laden SNF and DOX-laden SNF with different loading concentration and the DOX-PTX-co-loaded SNF with different ratios of DOX and PTX in the concentration range of 1–10000 nM were prepared and cultured with 4T1 cells (Fig. 5A–G). As shown in Fig. 5, the optimal synergistic action of DOX and PTX was achieved when the ratio of PTX/DOX was 4:1. The CI value in the optimized conditions was 0.52, significantly better than that of silk-based drug delivery systems. The density of DOX and PTX loaded on silk nanofibers and the total amount of drugs were also changed to reveal better drug composition for 4T1 cells (Fig. 5A–G). The IC_50_ values were also analyzed to clarify the optimal weight ratio and LC% of DOX and PTX co-loaded on SNF using GraphPad Prism software (Table S1-S3). The lowest IC_50_ value for PTX-laden SNF and DOX-laden SNF was achieved when the LC% of PTX and DOX were 4 % and 8 %, respectively (Tables S1 and S2). The lowest CI value was achieved when the ratio of PTX/DOX was 1:2 (Fig. 5H). Based on these results, the optimal ratio and LC% of PTX and DOX were determined to be 1:2, 4 %, and 8 %, respectively. The DOX-PTX co-laden SNF under the optimized conditions was then used to inhibit tumor growth *in vivo*.

### 3.3 Combination therapy platform composed of SNR and SNF

**Fig. 6:**
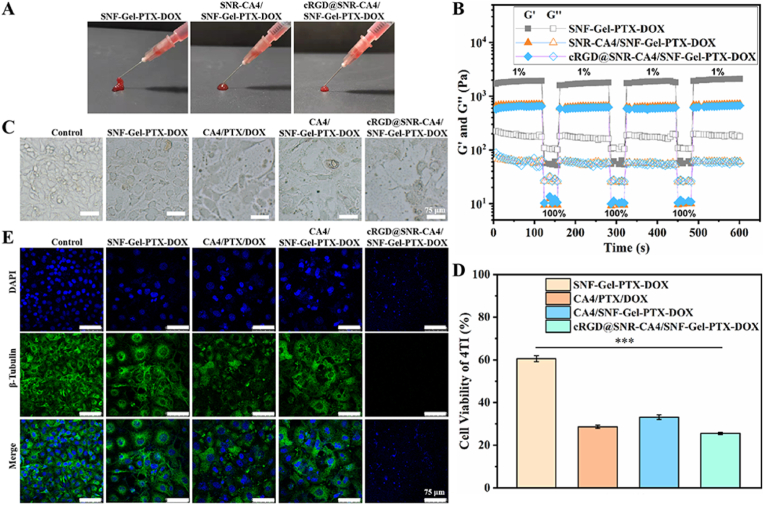
Syringe needle injection photograph (A) and rheological continuous step-strain test (B) for SNF-Gel-PTX-DOX, SNR-CA4/SNF-Gel-PTX-DOX, and cRGD@SNR-CA4/SNF-Gel-PTX-DOX. Cell morphology (C) and cell viability (D) of 4T1 cells incubated with samples for 48 h. (E) Immunofluorescence staining of β-Tubulin in HUVECs after incubation with samples for 48 h. Untreated cells were considered as control. DNA stained with DAPI (blue) and β-Tubulin marked green. The samples were as follows: SNF-Gel-PTX-DOX was PTX-DOX-co-laden SNF hydrogel, CA4/PTX/DOX was free CA4, PTX and DOX, CA4/SNF-Gel-PTX-DOX was free CA4 blended with SNF-Gel-PTX-DOX, cRGD@SNR-CA4/SNF-Gel-PTX-DOX was CA4-laden cRGD@SNR blended with SNF-Gel-PTX-DOX. Data presented as mean ± SD, n = 5, error bars indicate the SD, p-values were calculated using Student's *t*-test with a two-sided method, ∗∗∗P ≤ 0.001. (For interpretation of the references to colour in this figure legend, the reader is referred to the Web version of this article.)

A great challenge remains to fuse various drug carriers in a single injectable hydrogel system without compromising their biological function [13,40]. Our recent study indicated that SNF co-loaded with DOX and PTX assembled to injectable hydrogels, facilitating its application in local tumor treatment [23]. The drug-laden SNF hydrogel was a suitable matrix for incorporating other drug-laden carriers or functional components. Both CA4-laden SNR and PTX-DOX-co-laden SNF were dispersed in the aqueous environment and easily blended to form homogeneous hydrogels. All the composite hydrogels could be injected from 26 G needles and then solidified after the injection, confirming their injection property (Fig. 6A). The storage modulus at 1 % shear strain decreased from about 2000 Pa to 700 Pa after CA4-laden SNR blended with SNF hydrogels (Fig. 6B), which would improve the injecting and spreading capacity [44]. The hydrogel recovery rate was critical for the silk nanocarrier hydrogel to immobilize around tumor tissue and achieve long-term drug delivery. When the shear strain was switched repeatedly from 100 % to 1 %, all the drug-laden silk nanocarrier systems showed stable turnover between hydrogel and fluid (Fig. 6B). After the repeat switch four times, the recovery rate was still above 90 % at lower shear strain. The results confirmed that the drug carrier system could recover the hydrogel state after injection and then immobilize around tumor tissue, which would favor the long-term effect of the loaded drugs.

The beta-sheet rich structure of SNF and SNR endowed them with superior loading capacity for both hydrophobic and hydrophililc drugs. The improved stability could be achieved for the drugs loaded on SNF and SNR, favoring their clinical applications [22,23]. Mild physical mixing process at room temperature was used to develop the injectable drug cocktail hydrogels composed of CA4-laden cRGD@SNR and DOX-PTX co-laden SNF, which favored the activity maintenance of the drugs. Maintaining the respective biological function of various drug-laden carriers in the composite hydrogel system is pivotal to better combination therapy. Considering CA4 showed higher cytotoxicity to 4T1 and more efficiently restrained the β-tubulin polymerization of endothelial cells than SNR-CA4 (Fig. 3), free CA4 blended with PTX-DOX-laden SNF hydrogels was cultured with 4T1 cells and endothelial cells to confirm the targeting ability of the composite hydrogel system (Fig. 6C–E). Under the designed optimal ratio and amount of PTX and DOX, the survival rate of 4T1 cells decreased to about 60 % after 48 h (Fig. 6D). When free CA4 was blended with PTX-DOX-laden SNF hydrogels, significantly higher cytotoxicity was achieved, bringing the decrease in survival rate to 33 %. All the drugs maintained their bioactivity in the composite hydrogel system. Similar to previous studies [23,31], the medium containing free CA4, PTX, and DOX resulted in lower cell survival than the composite hydrogel group composed of free CA4 and PTX-DOX-laden SNF under the same drug concentrations, due to a higher concentration of free drugs in the medium. The targeting modification of SNR enhanced the cytotoxicity of the composite hydrogels. Although a lower dosage of free drugs existed in the medium containing the composite hydrogels, significantly lower cell survival existed compared with the CA4/PTX/DOX and CA4/SNF-Gel-DOX-PTX groups. The results suggested that the targeting ability of cRGD@SNR was maintained in the composite hydrogel system. To further confirm the targeting ability of the composite hydrogel system, the β-tubulin in HUVECs was stained and measured (Fig. 6E). Compared to the control group, the HUVECs cultured with SNF-Gel-DOX-PTX exhibited significantly lower secretion of β-tubulin, possibly since the loaded DOX and PTX were toxic to the endothelial cells. The composite hydrogel group composed of free CA4 and PTX-DOX-laden SNF further restrained the β-tubulin secretion due to the β-tubulin polymerization restraining function of CA4. As expected, free CA4, DOX, and PTX further restrained the β-tubulin secretion due to higher concentrations of free drugs in the medium. When CA4-laden cRGD@SNR were blended with DOX-PTX-laden SNF and used to culture with HUVECs, no significant β-tubulin was found, suggesting the effective inhibition of β-tubulin secretion. Thus, the cRGD@SNR reserved the targeting ability in the composite hydrogel system and achieved superior biological function to that of the cRGD@SNR-CA4 group and CA4/SNF-Gel-DOX-PTX group, which should be attributed to the synergistic effect of targeted CA4-laden SNR and DOX-PTX-laden SNF. Therefore, a drug cocktail platform was developed with different silk nanocarriers without the compromise of their respective biological activity. Based on the platform, different functions, such as targeting ability and co-delivery, could be designed actively and independently to optimize the combination therapy, which would provide efficient niches to inhibit tumor growth and metastasis.

### 3.4 In vivo tumor inhibition study

**Fig. 7:**
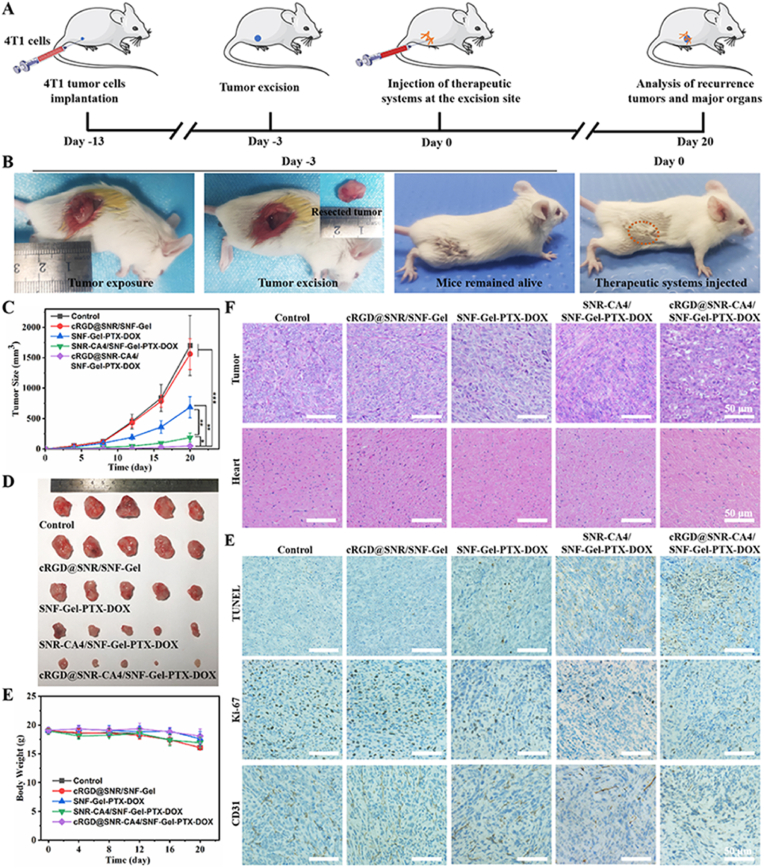
*In vivo* antitumor recurrence effect of normal saline (Control), cRGD@SNR/SNF-Gel, SNF-Gel-PTX-DOX, SNR-CA4/SNF-Gel-PTX-DOX, and cRGD@SNR-CA4/SNF-Gel-PTX-DOX in 4T1 tumor-bearing mice: (A) Schematic illustration of the postoperative recurrence and metastasis model for triple negative breast cancer, (B) photographs of 4T1 tumor-bearing mice with operative to resect primary tumor at day −3 and injected with therapeutic systems at the tumor excision site at day 0. (C) Tumor volumes as a function of time, (D) picture of excised tumors at 28 days, (E) body weight as a function of time, (F) H&E stained photomicrographs of tumor and heart tissue, 20 days after treatment. (G) TUNEL staining, Ki-67, and CD31 immunostaining of recurrent tumors after normal saline (Control), cRGD@SNR/SNF-Gel, SNF-Gel-PTX-DOX, SNR-CA4/SNF-Gel-PTX-DOX, and cRGD@SNR-CA4/SNF-Gel-PTX-DOX were injected at the 4T1 tumor excision site for 20 days. Blue stains indicate the cell nucleus stained with hematoxylin. Brown stains indicate apoptotic cells in TUNEL analysis, proliferating cells in Ki-67 immunostaining, and endothelial cells of tumor vessels in CD31 immunostaining, respectively. Data were presented as mean ± SD, n = 5. Error bars indicate the SD. One-way ANOVA combined with Bonferroni's multiple comparison Post Hoc test was used to evaluate statistical analysis, ∗P ≤ 0.05, ∗∗P ≤ 0.01, ∗∗∗P ≤ 0.001. (For interpretation of the references to colour in this figure legend, the reader is referred to the Web version of this article.)

Local synergistic therapy was a strategy for preventing postoperative tumor growth and metastasis [45,46]. Hydrogel is a promising platform for postoperative treatment with excellent therapeutic efficacy and favorable biosafety [47]. 4T1 cells are a highly aggressive mouse triple-negative breast cancer cell line. The growth and metastasis characteristics of the 4T1 tumor in BALB/c mice are similar to those of human breast cancer [48]. To assess the effect of the drug cocktail hydrogel on inhibiting postoperative recurrence and metastasis, a triple-negative breast cancer mouse model was developed with 4T1 cells. As shown in Fig. 7A and B, 4T1 cells were injected into the groin of the mice and cultured to induce tumor growth for approximately 10 days. When the size of the tumor increased to about 200 mm^3^, the tumors were excised. After the mice was fed for 3 days, cRGD@SNR/SNF-Gel, SNF-Gel-PTX-DOX, SNR-CA4/SNF-Gel-PTX-DOX and cRGD@SNR-CA4/SNF-Gel-PTX-DOX were injected to the tumor excision site to inhibit tumor recurrence, considering the CA4-laden SNR preferred to induce hemorrhage and restrain tumor growth than free CA4 *in vivo* (Fig. 4). Same volume of PBS was also injected into the tumor excision site and used as the blank control. The time was set as day 0. All the mice were raised to investigate the tumor recurrence behaviors. In the blank group, serious recurrence happened where the size of the recurrent tumor increased to above 1600 mm^3^ on day 20 (Fig. 7C and D). According to the guidelines of ethical requirements, the experiment was stopped at day 20. Similar to the blank group, the quick tumor growth was observed in the drug free cRGD@SNR/SNF-Gel group, confirming that both SNR and SNF cannot retard tumor recurrence. Recent studies revealed that a suitable ratio of DOX and PTX co-loaded on SNF could achieve the optimal combination chemotherapy [23]. The tumor growth was restrained when the rice was treated with the SNF-Gel-PTX-DOX hydrogel. On day 20, the tumor volume was 686 ± 173 mm^3^, which indicated the synergistic action of the loaded PTX and DOX. However, the continuous tumor growth in the SNF-Gel-PTX-DOX group suggested that more therapeutic intervention was required to improve tumor inhibition. After the SNR-CA4 was introduced to the SNF-Gel-PTX-DOX hydrogel, the combination therapy of CA4 and PTX/DOX showed a better therapeutic effect. The tumor size was only 188 ± 76 mm^3^ on day 20, less than a third of the tumors treated with SNF-PTX-DOX. The results revealed that the silk nanocarrier system was a feasible platform for holding different drugs, facilitating drug cocktail design. Compared to previous co-delivery systems [9,43,49], silk nanocarrier platform provided an option to optimize the specific biological function of the drugs loaded on different carriers. The SNR was modified with cRGDfK to introduce targeting ability, which enhanced the biological function of the loaded CA4. After 20 days, the tumor size in the cRGD@SNR-CA4/SNF-Gel-PTX-DOX group was only 48 ± 33 mm^3^, similar to that at day 0. The long-term inhibition of tumor recurrence was achieved through optimizing the targeting effects of CA4 on tumor vessels, and also the combination chemotherapy of PTX and DOX in the platform. The balance between therapeutic efficiency and side effects of different antitumor drugs usually limits their clinical application. *In situ* local delivery system is considered a preferable strategy for enhancing the therapeutic effect and alleviating the adverse effects of drugs simultaneously [50]. As expected, the drugs loaded on silk nanocarriers had no evident side effects. The weight of the mice treated with different drug-laden hydrogels only reduced slightly after 20 days, where the treated group and the blank group had no significant difference (Fig. 7E). Previous studies revealed that all the used drugs (CA4, DOX, and PTX) had potential damage to cardiac tissue in clinical applications [51,52]. To further clarify the side effect of the drugs in our drug cocktail hydrogel system, the sections of tumor and heart were collected from different treated rice and stained with H&E at day 20 to visualize the microstructure changes (Fig. 7F). In the blank control group and drug free cRGD@SNR/SNF-Gel group, the tumor tissues maintained dense structure with obvious cell atypia, confirming their typical progression state. When the tumors were treated with SNF-Gel-PTX-DOX, SNR-CA4/SNF-Gel-PTX-DOX, and cRGD@SNR-CA4/SNF-Gel-PTX-DOX hydrogels, respectively, the cell density of tumor tissues gradually decreased, and the ratio of dead tumor cells increased, which was fully consistent with the tumor recurrence results. Since CA4, DOX and PTX tend to attack cardiac tissue, the heart sections of different groups were collected and stained to evaluate the systemic toxicity of the hydrogel delivery system. All the heart sections in different groups had no significant pathological changes, indicating that the hydrogel delivery system significantly reduced the side effects of all the drugs. Further systemic toxicity evaluations including liver and kidney will be continued in our following study. Thus, the targeted effect on tumor vessels and combination chemotherapy of PTX and DOX on tumor tissues were fused and optimized in our silk nanocarrier hydrogels, which realized the effective long-term inhibition of breast tumor recurrence.

To clarify the specific function of different drugs in the system, the tumor sections collected on day 20 were stained with TUNEL, Ki-67, and CD31, respectively (Fig. 7G). Both the blank control and drug-free cRGD@SNR/SNF-Gel groups had plenty of proliferating tumor cells and neovascular structures without apoptotic tumor cells. When the tumors were treated with the SNF-Gel-PTX-DOX hydrogel, the apoptotic tumor cells appeared, followed by a decrease in the proliferating tumor cells. The results revealed that the co-delivery of PTX and DOX induced tumor apoptosis. Rich vascular structures were still maintained in the SNF-Gel-PTX-DOX group, implying that the loaded DOX and PTX cannot act on the tumor vessels. After the introduction of CA4-laden SNR in the SNF-Gel-PTX-DOX hydrogel, the vascular density in the tumor tissue significantly reduced due to the vascular inhibition function of CA4. Thanks to the cooperative action of the loaded CA4 and DOX/PTX, a higher ratio of apoptotic cells appeared in the tumor tissue. The vascular targeting modification of SNR further enhanced the damage to the tumor vessels. Minority blood vessels could be found in the tumor tissue treated with the cRGD@SNR-CA4/SNF-Gel-PTX-DOX hydrogels. The tumor tissues were mainly composed of apoptotic cells, which confirmed that the tumors kept an apoptotic state after 20 days. Thus, the optimized DOX and PTX effectively kill cancer cells while the CA4 loaded on the cRGD@SNR destroyed the tumor vessels, resulting in the best inhibition of postoperative recurrence.

**Fig. 8:**
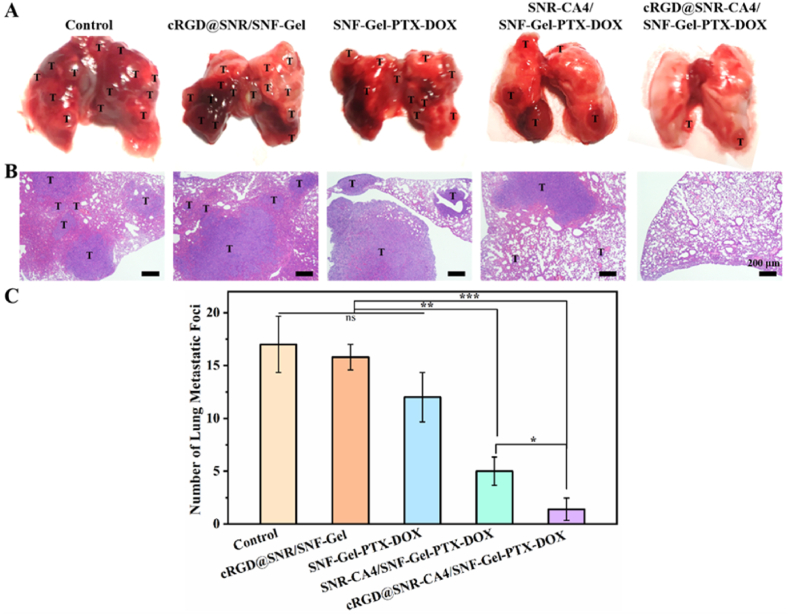
Lung metastasis of murine breast tumors after different treatment groups: (A) representative lung tissue photographs, (B) H&E-stained lung sections collected after the animals were treated for 20 days, (C) number of lung metastatic foci in each group. Metastatic tumors in lung tissues are marked with a “T”. The treatment groups are saline (Control), cRGD@SNR/SNF-Gel, SNF-Gel-PTX-DOX, SNR-CA4/SNF-Gel-PTX-DOX and cRGD@SNR-CA4/SNF-Gel-PTX-DOX. Data presented as mean ± SD, n = 5, error bars indicated the SD, p-values were calculated using Student's *t*-test with two-sided method, ns > 0.05, ∗P ≤ 0.05, ∗∗P ≤ 0.01, ∗∗∗P ≤ 0.001.

Tumor metastasis is a main reason for the failure of tumor resection. The lung is prone to metastasis by 4T1 tumors [53]. The tumor metastasis in lung tissue was investigated when the mice were treated with different drug-laden hydrogels for 20 days (Fig. 8). In the blank control and drug-free hydrogel group, the lesion number in the lung tissue was 17 and 16, indicating serious tumor metastasis (Fig. 8A–C). The tumor vascular network could accelerate the metastasis. Twelve lesions still existed in the lung tissue when the mice were treated with the SNF-Gel-PTX-DOX hydrogel, since the combination chemotherapy of DOX and PTX never destroyed the vascular vessels effectively. The CA4-laden SNR was introduced to the SNF-Gel-PTX-DOX hydrogel to destroy the tumor vascular network, and then also restrained the metastasis significantly. The lesions in the CA4-SNR-SNF-Gel-PTX-DOX group were decreased to 5, which is a drop to above 2 folds than the SNF-Gel-PTX-DOX group. The targeting modification of SNR improved the effect of CA4 on the tumor vessel, further strengthening the metastasis inhibition. Only two small lesions were found in the lung tissue in the cRGD@SNR-CA4/SNF-Gel-PTX-DOX. The present results suggested that tumor angiogenesis was the main reason for tumor metastasis. The H&E stained section confirmed that most of lung tissues have been occupied by tumor cells in the blank, cRGD@SNR/SNF-Gel and SNF-Gel-PTX-DOX groups while almost all the lung tissue maintained normal state in the cRGD@SNR-CA4/SNF-Gel-PTX-DOX group (Fig. 8B). Both targeting modification of SNR and the co-delivery of DOX and PTX on the SNF enhanced the biological activity of different drugs, which then improved the inhibition of tumor recurrence and metastasis, superior to most of combination therapy systems reported previously [13,16,26,27].

## 4 Conclusions

The SNR with a size of about 80 nm was modified with cRGDfK to achieve targeting ability to tumor blood vessels. Hydrophobic CA4 was loaded on the modified nanorods and blended with silk nanofibers co-loaded with DOX and PTX to form an injectable composite hydrogel. Both the targeting ability and the combination chemotherapy of DOX and PTX could be tuned in the composite hydrogels to enhance the biological activity of different drugs. The drug cocktail hydrogels were injected into the tumor excision site, achieving effective long-term inhibition of recurrence and metastasis. Our present study provided an injectable platform to design specific biological functions based on multiple needs of tumor treatment, which has a promising future in combination therapy with optimal therapeutic efficiency.

## CRediT authorship contribution statement

**Liying Xiao:** Writing – original draft, Methodology, Investigation, Conceptualization. **Jianwen Hou:** Methodology, Data curation. **Hongxiang Liu:** Methodology, Investigation. **Qiang Lu:** Writing – review & editing, Supervision, Project administration, Investigation.

## Declaration of competing interest

The authors declare that they have no known competing financial interests or personal relationships that could have appeared to influence the work reported in this paper.

## Data Availability

Data will be made available on request.
