# Supplementary material for: Targeting drug cocktail hydrogel platform for inhibiting tumor growth and metastasis
Source: Mater Today Bio. 2025 Apr 23;32:101798. doi: 10.1016/j.mtbio.2025.101798 (PMC12059701; doi:10.1016/j.mtbio.2025.101798)
Supplement: Multimedia component 1 [file mmc1.doc]

**Appendix. Supplementary data**

Targeting drug cocktail hydrogel platform for inhibiting tumor growth and metastasis

Liying Xiao^a,b^, Jiangwen Hou^c^, Hongxiang Liu^d^, Qiang Lu^a,d,*^

^a^Institutes for Translational Medicine, Soochow University, Suzhou 215123, People’s Republic of China

^b^National Engineering Laboratory for Modern Silk & Collaborative Innovation Center of Suzhou Nano Science and Technology, Soochow University, Suzhou 215123, People’s Republic of China

^c^Department of Trauma Orthopedics, The Second People’s Hospital of Lianyungang Affiliated to Bengbu Medical College, Lianyungang 222023, People’s Republic of China

^d^Department of Orthopedics, The Second Affiliated Hospital of Soochow University, Soochow University, Suzhou 215000, People’s Republic of China

Corresponding author:

Qiang Lu, Tel: (+86)-512-67061649; E-mail: lvqiang78@suda.edu.cn

**
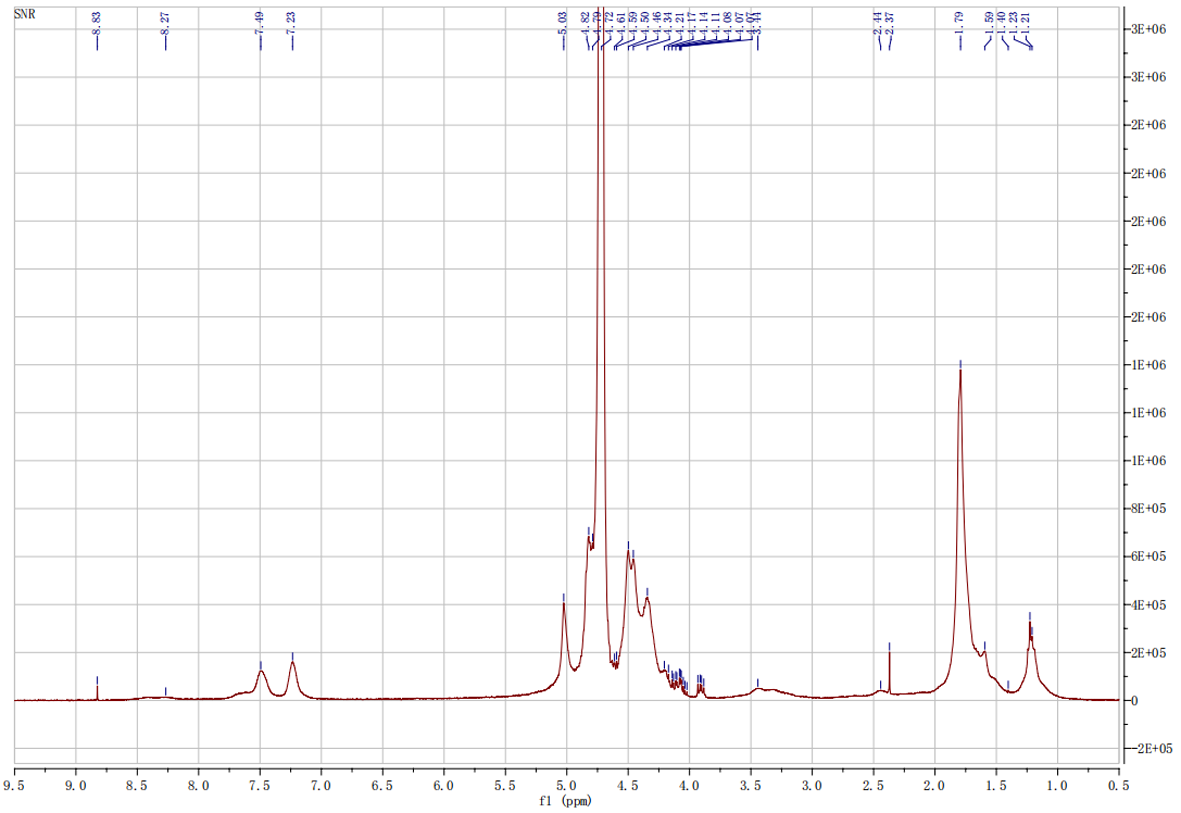
**

Fig. S1. ^1^H NMR spectra of SNR. The SNR was dissolved in deuterium oxide (D20) containing 9.3 M LiBr, and ^1^H NMR spectra were determined in an AVANCE NEO NMR system at 400 MHz (Bruker, Karlsruhe, Germany). The peak at 4.72 ppm was attributed to the solvent (D_2_0). The benzene ring group attributed the peaks between 7.1 and 8.6 ppm.

**
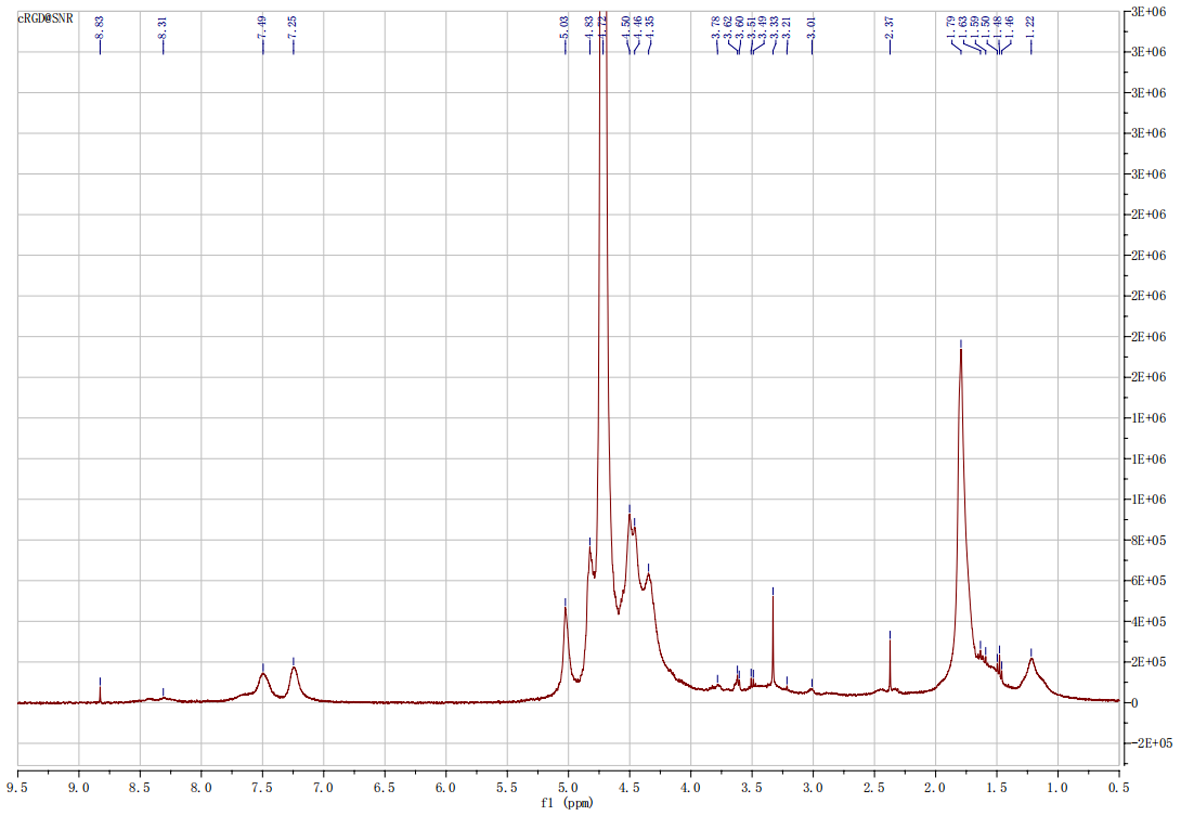
**

Fig. S2. ^1^H NMR spectra of cRGD@SNR. The cRGD@SNR was dissolved in deuterium oxide (D20) containing 9.3 M LiBr, and ^1^H NMR spectra were determined in an AVANCE NEO NMR system at 400 MHz (Bruker, Karlsruhe, Germany). The peak at 4.72 ppm was attributed to the solvent (D_2_0). The benzene ring group attributed the peaks between 7.1 and 8.6 ppm.

**Table S1.** IC_50_ values of Free PTX and PTX-laden SNF with 2%, 4%, and 8% of PTX were loaded against 4T1 cells after 48 hours of incubation, analyzed using GraphPad Prism software based on the cell viability.

|  | Free PTX | SNF-Gel-PTX_2%_ | SNF-Gel-PTX_4%_ | SNF-Gel-PTX_8%_ |
| --- | --- | --- | --- | --- |
| IC_50_ of PTX (nM) | NA | 5236 | 9871 | 13275 |

**Table S2.** IC_50_ values of Free DOX and DOX-laden SNF with 2%, 4%, and 8% of DOX were loaded against 4T1 cells after 48 hours of incubation, analyzed using GraphPad Prism software based on the cell viability.

|  | Free DOX | SNF-Gel-DOX_2%_ | SNF-Gel-DOX_4%_ | SNF-Gel-DOX_8%_ |
| --- | --- | --- | --- | --- |
| IC_50_ of DOX (nM) | 230 | 233 | 201 | 172 |

**Table S3.** IC_50_ values of Free PTX/DOX and SNF-Gel-PTX-DOX with the ratio of PTX/DOX at 4:1，2:1，1:1，1:2, and 1:4 against 4T1 cells after 48 hours of incubation, analyzed using GraphPad Prism software based on the cell viability.

| Treatment | IC50 of PTX (nM) | IC50 of DOX (nM) |
| --- | --- | --- |
| Free PTX/DOX with the ratio of PTX/DOX at 4:1 | 476 | 119 |
| Free PTX/DOX with the ratio of PTX/DOX at 2:1 | 249 | 125 |
| Free PTX/DOX with the ratio of PTX/DOX at 1:1 | 149 | 149 |
| Free PTX/DOX with the ratio of PTX/DOX at 1:2 | 67 | 134 |
| Free PTX/DOX with the ratio of PTX/DOX at 1:4 | 37 | 149 |
| SNF-Gel-PTX-DOX with the ratio of PTX/DOX at 4:1 | 616 | 154 |
| SNF-Gel-PTX-DOX with the ratio of PTX/DOX at 2:1 | 340 | 170 |
| SNF-Gel-PTX-DOX with the ratio of PTX/DOX at 1:1 | 161 | 161 |
| SNF-Gel-PTX-DOX with the ratio of PTX/DOX at 1:2 | 67 | 134 |
| SNF-Gel-PTX-DOX with the ratio of PTX/DOX at 1:4 | 40 | 159 |
